# Book Review

**Published:** 1874-02

**Authors:** D. C. Hawxhurst


### Editorial.

### BOOK REVIEW.

*Dental Caries and its Causes; An Investigation into the Influence of Fungi in the Destruction of Teeth*: By DRS. LEBER AND ROTTENSTEIN. Translated by Thomas H. Chandler, Professor of Mechanical Dentistry in the Dental School of Harvard University. With illustrations. Philadelphia: Lindsay and Blakiston, 1873.

During the last few years a vague idea of animalculæ in and about the teeth has been floating among the masses of the people. The idea originated, no doubt, in actual investigation with the lens. At different times parasites have been variously described by several French and German writers; but never, I believe, with so great a degree of particularity, previous to the appearance of this book. What was needed was some accurate portrayal of the character of the organism itself, in such terms as would enable other observers to find it, along with such a description of the methods of research employed as might enable other investigators to verify the statements made about it.

These things have been thoroughly accomplished by the authors of "Dental Caries and its Causes."

And one thing more they have done: they have investigated the precise part which the parasite plays in the progress of caries. Other writers have mostly contented themselves with demonstrating the existence of a living organism, leaving it still an open question, whether it were a mere accident of the oral cavity or an active cause of morbific results. The authors of this work have described with great exactness the precise action which the *leptothrix buccalis* has upon dentine, with many details of the destructive process, which must have cost them long-continued labor. The methods employed in these researches are so fully detailed that anybody can verify for himself.

It is also to be remarked that "Dental Caries and its Causes" is not alone an inquiry into the part which the *leptothrix* plays in the production of caries, but contains also many acute observations on the questions of vital and chemical action. The progressive changes of decomposing dentine are accurately set forth and the *philosophy* of decay is discussed.

Few will read this neat little volume of 99 pages without being convinced that the *leptothrix* exerts a powerful influence in destroying dentine after its integrity has been impaired by partial decalcification.

A single quotation will serve to introduce these authors to the reader :

"We often observe in sections islets which are filled with the fine granular matter of the fungus. (Plate II. Fig. 6.) The origin of these islets is easily explained by the direction of the sections, which cross a conduit filled with *leptothrix* and which, in consequence, offer the image of an isolated islet. We see, moreover, other contents, which correspond with the surface. Up to the present time observers have confined themselves to proving the presence of the *leptothrix* in the carious cavities of the tooth, but without assigning it any influence in the work of decomposition. They have not noticed the introduction of the *leptothrix* into the softened substance of the dentine; and its presence in the carious cavity has consequently been considered as accidental and without importance. According to our observation, we can not refuse to admit that the proliferation of the fungus plays an important part in the decomposition of the dental tissue.

What explains the ignorance of authors on this point is, that they confounded the vegetable granulations with the organic matters in decomposition and that the elements proper to *leptothrix* passed unperceived."

The volume is illustrated by elegant plates, which render the precise meaning of the authors clear and may act as aids to private investigators who would verify for themselves the remarkable phenomena pointed out by the distinguished authors. The evident fairness of statement and fullness of detail are in themselves pledges of merit. I cannot but believe this work should be in the hands of every practitioner who would promptly follow out every clue to a solution of the question of dental caries. D. C. HAWXHURST.

